# Posttranslational Targeting of a Recombinant Protein Promotes Its Efficient Secretion into the Escherichia coli Periplasm

**DOI:** 10.1128/AEM.00671-19

**Published:** 2019-06-17

**Authors:** A. Jimmy Ytterberg, Roman A. Zubarev, Thomas Baumgarten

**Affiliations:** a Chemistry I Division, Department of Medical Biochemistry and Biophysics, Karolinska Institute, Stockholm, Sweden; b Rheumatology Unit, Department of Medicine, Karolinska Institute, Stockholm, Sweden; c Division of Glycoscience, Department of Chemistry, KTH Royal Institute of Technology, AlbaNova University Centre, Stockholm, Sweden; University of Tartu

**Keywords:** *Escherichia coli*, periplasm, protein secretion, proteomics, recombinant protein production

## Abstract

Many recombinant proteins that are produced in Escherichia coli have to be targeted to the periplasm to be functional. N-terminal signal peptides can be used to direct recombinant proteins to the membrane-embedded Sec translocon, a multiprotein complex that translocates proteins across the membrane into the periplasm. We have recently shown that the cotranslational targeting of the single-chain variable antibody fragment BL1 saturates the capacity of the Sec translocon leading to impaired translocation of secretory proteins and protein misfolding/aggregation in the cytoplasm. In turn, protein production yields and biomass formation were low. Here, we study the consequences of targeting BL1 posttranslationally to the Sec translocon. Notably, the posttranslational targeting of BL1 does not saturate the Sec translocon capacity, and both biomass formation and protein production yields are increased. Analyzing the proteome of cells producing the posttranslationally targeted BL1 indicates that the decreased synthesis of endogenous secretory and membrane proteins prevents a saturation of the Sec translocon capacity. Furthermore, in these cells, highly abundant chaperones and proteases can clear misfolded/aggregated proteins from the cytoplasm, thereby improving the fitness of these cells. Thus, the posttranslational targeting of BL1 enables its efficient production in the periplasm due to a favorable adaptation of the E. coli proteome. We envisage that our observations can be used to engineer E. coli for the improved production of recombinant secretory proteins.

**IMPORTANCE** The bacterium Escherichia coli is widely used to produce recombinant proteins. To fold properly, many recombinant proteins have to be targeted to the E. coli periplasm, but so far the impact of the targeting pathway of a recombinant protein to the periplasm has not been extensively investigated. Here, we show that the targeting pathway of a recombinant antibody fragment has a tremendous impact on cell physiology, ultimately affecting protein production yields in the periplasm and biomass formation. This indicates that studying the targeting and secretion of proteins into the periplasm could be used to design strategies to improve recombinant protein production yields.

## Abstract (precis)

The bacterium Escherichia coli is widely used to produce recombinant proteins. To fold properly, many recombinant proteins have to be targeted to the E. coli periplasm, but so far the impact of the targeting pathway of a recombinant protein to the periplasm has not been extensively investigated. Here, we show that the targeting pathway of a recombinant antibody fragment has a tremendous impact on cell physiology, ultimately affecting protein production yields in the periplasm and biomass formation. This indicates that studying the targeting and secretion of proteins into the periplasm could be used to design strategies to improve recombinant protein production yields.

## INTRODUCTION

The Gram-negative bacterium Escherichia coli is frequently used to produce recombinant proteins (1, 2). The periplasm of E. coli contains the Dsb system that catalyzes the formation of disulfide bonds, thereby enabling correct folding of recombinant proteins, such as antibody fragments and many peptide hormones (3–5). In addition, in the periplasm, various chaperones can facilitate the correct folding of recombinant proteins (6). Furthermore, it is easier to isolate recombinant proteins from the periplasm rather than from whole cell lysates (6).

To reach the periplasm, recombinant secretory proteins have to be translocated across the inner membrane. Most proteins are secreted across the inner membrane in an unfolded state via the Sec translocon, which is an evolutionarily conserved multiprotein complex that facilitates the biogenesis of both secretory and membrane proteins (7). Targeting of proteins to the Sec translocon is facilitated by their N-terminal signal peptides, and a recombinant protein destined for the periplasm is equipped with such a targeting signal (8). Depending on the signal peptide, a recombinant secretory protein is either routed into the cotranslational signal recognition particle (SRP)-dependent targeting pathway or directed in a posttranslational SecA/SecB-dependent manner to the Sec translocon (9–11). Upon protein translocation, the signal peptide is cleaved off by leader peptidase (12).

Recently, we found that the production of recombinant proteins that are equipped with the DsbA signal peptide, which directs proteins into the cotranslational targeting pathway, leads to the saturation of the Sec translocon capacity (13–15). As a consequence, protein translocation is impaired, leading to the accumulation of precursors of secretory proteins in the cytoplasm and induction of the σ^32^ response due to protein misfolding/aggregation in the cytoplasm (14). In addition, it has been demonstrated that the production of membrane proteins, which also follow the cotranslational targeting pathway, hampers protein translocation due to the saturation of the Sec translocon capacity, resulting in consequences that are similar to what is observed when cotranslationally targeted secretory proteins are produced (16, 17).

On the other hand, recombinant proteins are routinely equipped with signal peptides that direct them posttranslationally to the Sec translocon, such as the OmpA signal peptide (18). However, the consequences of producing such proteins have not been investigated in detail, making it difficult to improve the production of such proteins. Here, we compare the consequences of directing a model single-chain variable antibody fragment either cotranslationally or posttranslationally to the Sec translocon by using a proteomics approach.

## RESULTS

### Posttranslational targeting of the scFv BL1 does not impair protein translocation.

Previously, we studied the consequences of producing the single-chain variable antibody fragment (scFv) BL1 fused to the DsbA signal peptide, which facilitates cotranslational targeting of secretory proteins to the Sec translocon (14, 15). Here, we fused the scFv BL1 genetically to the OmpA signal peptide and compared the consequences of producing the posttranslationally targeted OmpA-BL1 to the well-characterized consequences of producing the cotranslationally targeted DsbA-BL1 (13, 14).

First, to test if using different signal peptides affects protein production kinetics due to potential differences in the translation initiation region of the recombinant transcript, we fused the OmpA signal peptide and the DsbA signal peptide genetically to superfolder green fluorescent protein (sfGFP) and monitored fluorescence over time. Notably, the production kinetics of both recombinant proteins were alike, indicating that replacing the DsbA signal peptide with the OmpA signal peptide does not affect protein production kinetics (see Fig. S1 in the supplemental material). This is also in line with very similar free energy values for the predicted mRNA structure around the translation initiation region (see Fig. S1) (19).

**FIG 1:**
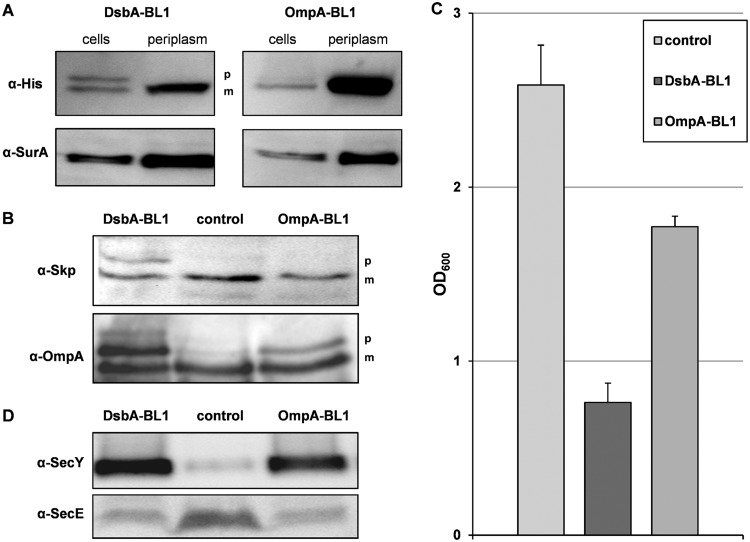
Impact of the targeting pathway of the produced scFv BL1. Production of either DsbA-BL1 or OmpA-BL1 was induced with IPTG in Tuner(DE3) cells for 4 h. Tuner(DE3) cells harboring an empty expression vector were used as control. (A) Levels of the precursor (p) and the mature form (m) of the scFv BL1 were monitored using SDS-PAGE followed by immunoblotting in whole cells and the periplasmic fraction using an α-His antibody recognizing the C-terminal His tag of the scFv BL1. The periplasmic chaperone SurA was used as a marker to monitor the efficiency of isolating the periplasmic fraction. The proper folding of the scFv BL1 was assessed using a dot blot assay (see Fig. S2 in the supplemental material). (B) The presence of the precursor (p) and mature form (m) of Skp and OmpA was probed using SDS-PAGE followed by immunoblotting. (C) Biomass formation was monitored by measuring the OD_600_. Biomass formation was also monitored over time (see Fig. S3). (D) The accumulation levels of the Sec translocon components SecY and SecE were monitored by means of immunoblotting.

It has been shown before that the production of a recombinant protein that is targeted to the periplasm can saturate the capacity of the Sec translocon, leading to the accumulation of precursor forms of secretory proteins in the E. coli cytoplasm (13, 14). The production of DsbA-BL1 impaired protein translocation dramatically as judged from the accumulation of the precursor form of the secretory scFv BL1 (Fig. 1A, upper left). Moreover, the secretion of the endogenous secretory proteins Skp and OmpA was impaired upon the production of DsbA-BL1 (Fig. 1B). In contrast, we found that if the scFv BL1 was posttranslationally targeted to the Sec translocon, no precursor of the scFv BL1 or Skp was detected and only some precursor of OmpA appears to be present (Fig. 1A, upper right, and Fig. 1B). This indicates that the posttranslational targeting of the secretory scFv BL1 does not saturate the capacity of the Sec translocon, resulting in improved production yields of the scFv BL1 in the periplasm and increased biomass formation (Fig. 1A and C).

Our previous study demonstrated that producing DsbA-BL1 results in increased levels of the core Sec translocon component SecY and decreased levels of the other core Sec translocon component, SecE (14). We speculated that this different ratio of SecY and SecE results in a decreased availability of functional Sec translocons, thereby lowering the translocation capacity of the cell (14). To test whether the production of OmpA-BL1 does not alter the ratio of SecY and SecE and therefore results in more functional Sec translocons, we compared accumulation levels of SecY and SecE in cells producing DsbA-BL1, OmpA-BL1, or no recombinant protein. For the production of both DsbA-BL1 and OmpA-BL1, we observed increased levels of SecY and decreased levels of SecE, indicating that these changes are independent of the targeting pathway (Fig. 1D). Since this does not explain why the Sec translocon capacity appears to be not saturated upon the production of OmpA-BL1, we decided to characterize the consequences of producing the posttranslationally targeted OmpA-BL1 using a proteomics approach.

### Consequences of producing the posttranslationally targeted scFv BL1.

To study the consequences of producing a posttranslationally targeted recombinant secretory protein, we analyzed the proteome of cells producing OmpA-BL1 by mass spectrometry. In addition, we also analyzed the proteome of cells producing DsbA-BL1, where the Sec translocon capacity is known to be saturated. As a reference, cells with an empty expression vector were used. Proteins were first extracted from cell lysates using acetone precipitation, subsequently solubilized, and digested in solution. The digested material was analyzed using label-free mass spectrometry.

**FIG 2:**
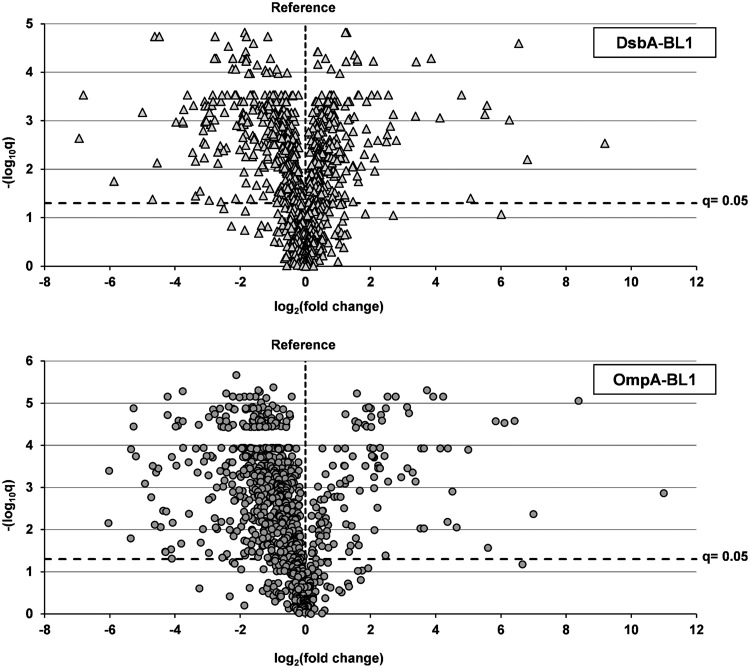
Impact of the targeting pathway of the produced scFv BL1 on the proteome composition. Production of either DsbA-BL1 or OmpA-BL1 was induced with IPTG in Tuner(DE3) cells. Tuner(DE3) cells harboring an empty expression vector were used as a reference. The proteomes of aforementioned cells were analyzed using label-free mass spectrometry. The *q* values, plotted as −log_10_ on the *y* axis, are plotted against the relative fold changes in protein abundance, plotted as log_2_ on the *x* axis. The horizontal interrupted line indicates the significance threshold; *q* values below this line represent changes that are not considered to be significant, whereas *q* values above this line represent changes that are considered to be significant. The upper panel illustrates the changes in the proteome of cells producing DsbA-BL1 (see Table S1 in the supplemental material). The triangles represent all identified and quantified proteins in these cells. The lower panel illustrates the changes in the proteome of cells producing OmpA-BL1 (see Table S2). The circles represent all identified and quantified proteins in these cells.

In total, 1,030 proteins could be identified and quantified in each sample, and the accumulation level of a protein was considered to be significantly changed if the false-discovery rate (*q*)-corrected *P* value was lower than 0.05 (see Tables S1 and S2 in the supplemental material). In cells producing DsbA-BL1, the levels of 662 proteins were affected, whereas in cells producing OmpA-BL1, the accumulation levels of 815 proteins were changed (Fig. 2; see also Tables S1 and S2). Thus, although the production of OmpA-BL1 does not lead to a saturation of the Sec translocon capacity and only moderately impairs biomass formation, it has a tremendous impact on the proteome.

**FIG 3:**
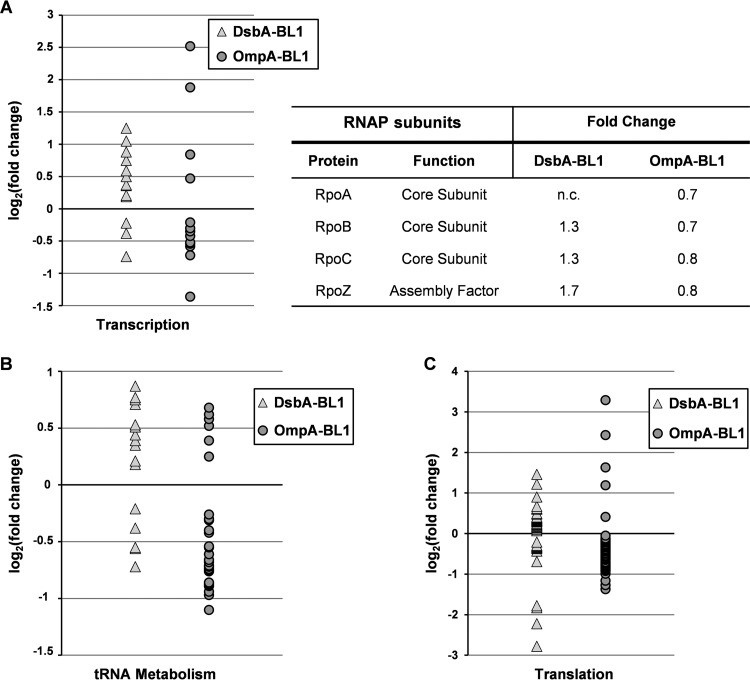
Impact of the targeting pathway of the produced scFv BL1 on protein synthesis. Production of either DsbA-BL1 or OmpA-BL1 was induced with IPTG in Tuner(DE3) cells. Tuner(DE3) cells harboring an empty expression vector were used as a reference. The proteomes of aforementioned cells were analyzed using label-free mass spectrometry, and the fold changes in the abundance of proteins involved in transcription, tRNA metabolism, and translation were analyzed (see Table S3 in the supplemental material). The triangles represent proteins that were significantly changed in cells producing DsbA-BL1. The circles represent proteins that were significantly changed in cells producing OmpA-BL1. (A, left) Fold changes of proteins that are involved in transcription are plotted as log_2_(fold change) on the *y* axis. (Right) Accumulation levels of the subunits of the RNA polymerase are compared (n.c., not changed). (B) Fold changes of proteins that are involved in tRNA metabolism are plotted as log_2_(fold change) on the *y* axis. (C) Fold changes of proteins that are involved in translation are plotted as log_2_(fold change) on the *y* axis.

By analyzing how the accumulation levels of membrane and secretory proteins are affected upon the production of OmpA-BL1, we found that about 75% of these proteins showed reduced accumulation levels (Fig. 2; see also Table S2 in the supplemental material). This may signify that protein synthesis and protein turnover are affected in these cells. Therefore, we looked specifically at all proteins that are involved in both processes, and we found that in cells producing OmpA-BL1, the vast majority of proteins associated with transcription (e.g., RpoB), loading of tRNAs, and translation (e.g., ribosomal proteins) had decreased accumulation levels (Fig. 3; see also Table S3). In conclusion, it seems likely that a decreased synthesis of endogenous membrane and secretory proteins prevents the Sec translocon from being saturated upon the production of OmpA-BL1.

In contrast, in cells producing DsbA-BL1, the majority of proteins that are involved in transcription, loading of tRNAs, and translation had increased accumulation levels, and it is likely that the enhanced synthesis of membrane and secretory proteins aggravates the saturation of the Sec translocon (Fig. 3; Table S3).

**FIG 4:**
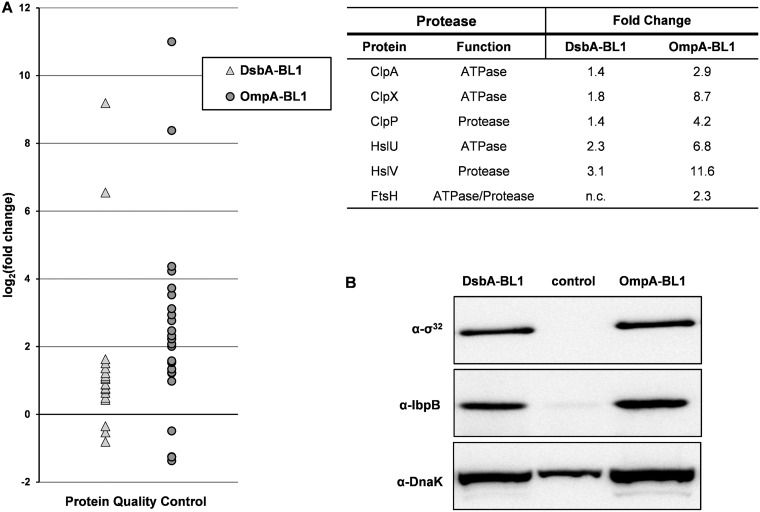
Impact of the targeting pathway of the produced scFv BL1 on protein degradation and quality control. Production of either DsbA-BL1 or OmpA-BL1 was induced with IPTG in Tuner(DE3) cells. Tuner(DE3) cells harboring an empty expression vector were used as a reference. The proteomes of aforementioned cells were analyzed using a combination of label-free mass spectrometry and immunoblotting. (A) Mass spectrometry was used to determine fold changes in the abundance of proteins that are involved in protein quality control. (Left) The fold changes of these proteins are plotted as log_2_(fold change) on the *y* axis (see Table S3 in the supplemental material). The triangles represent proteins that were significantly changed in cells producing DsbA-BL1. The circles represent proteins that were significantly changed in cells producing OmpA-BL1. (Right) Accumulation levels of the ATPase subunits and the protease subunits of the main E. coli proteases are compared (n.c., not changed). (B) Accumulation levels of σ^32^ and the σ^32^-regulated proteins IbpB and DnaK were monitored by means of immunoblotting.

Finally, we focused on proteins that are involved in protein folding and degradation in the cytoplasm. We found that the accumulation levels of many chaperones and proteases were increased upon the production of both OmpA-BL1 and DsbA-BL1 (Fig. 4; Table S3). However, the accumulation levels of these proteins were increased much more upon the production of OmpA-BL1 than they were increased when DsbA-BL1 was produced. This suggests that misfolded and incorrectly targeted proteins can be cleared faster from the cytoplasm of cells producing OmpA-BL1, thereby potentially improving cell fitness.

Taken together, the production of the posttranslationally targeted scFv BL1 leads to the decreased synthesis of endogenous membrane and secretory proteins, thereby preventing a saturation of the Sec translocon, and highly abundant chaperones and proteases may eliminate misfolded/aggregated proteins efficiently from the cytoplasm, thereby improving the cell fitness.

## DISCUSSION

In E. coli, many recombinant proteins, in particular the ones containing disulfide bonds, are targeted to the periplasm (4–6). We have previously shown that the production of recombinant secretory proteins that are cotranslationally targeted to the Sec translocon severely impairs protein translocation, resulting in low protein production yields and low biomass formation (13, 14). We complemented our previous work with this study, where we investigated the consequences of producing a recombinant secretory protein that is posttranslationally targeted to the Sec translocon. Surprisingly, we found that the posttranslational targeting of the scFv BL1 does not significantly hamper protein translocation, if at all.

Previously, we observed that the ratio of the core Sec translocon components SecY and SecE is affected upon the production of the cotranslationally targeted scFv BL1, and we proposed that this leads to the decreased availability of functional Sec translocons, thereby lowering the capacity of the Sec translocon (14). However, here we show that accumulation levels of SecY and SecE are affected in the same way if the scFv BL1 is targeted posttranslationally to the Sec translocon and that under this condition the capacity of the Sec translocon is not saturated. Therefore, it remains elusive how the altered accumulation levels of SecY and SecE affect the protein translocation capacity of the cell.

The observation that the capacity of the Sec translocon is not saturated upon the production of the posttranslationally targeted scFv BL1 may be explained by the decreased levels of proteins involved in protein synthesis resulting in the reduced synthesis of endogenous membrane and secretory proteins. For the production of membrane and secretory proteins, it has been shown that decreasing the production rate of the recombinant protein by lowering the expression intensity of the recombinant gene can prevent a saturation of the Sec translocon and increases protein production yields and biomass formation (13, 17, 20, 21). Here, we propose that the reduced synthesis of endogenous membrane and secretory proteins prevents a saturation of the Sec translocon capacity, thereby increasing protein production yields and biomass formation.

Some of the observed changes in the proteome of cells producing OmpA-BL1 appear to be reminiscent of the stringent response (22). However, based on our proteomic data, we could not find strong indications for an induction of the stringent response; e.g., proteins involved in the synthesis of certain amino acids did not show increased accumulation levels. Therefore, it appears likely that the observed changes are not derived from the activation of a single regulon but are the result of a complex regulatory network potentially acting on the transcriptional and posttranscriptional level.

The induction of the σ^32^ response has been observed before for the production of recombinant proteins as a consequence of protein misfolding/aggregation in the cytoplasm and appears to be generic (14, 16). Nevertheless, when the produced scFv BL1 was targeted posttranslationally to the Sec translocon, accumulation levels of many chaperones and proteases were further increased compared to the production of the scFv BL1 that was cotranslationally targeted to the Sec translocon. It seems likely that this increased abundance of chaperones and proteases facilitates that misfolded/aggregated proteins are cleared more efficiently from the cytoplasm, thereby improving the cell fitness.

Taken together, the production of the posttranslationally targeted scFv BL1 does not saturate the capacity of the Sec translocon due to the reduced synthesis of endogenous membrane and secretory proteins. This indicates that besides modulating the production rate of a recombinant secretory protein, the reduced synthesis of endogenous secretory proteins can also lower the load on the Sec translocon, thereby increasing protein production yields and biomass formation. We envision that manipulating the synthesis of endogenous secretory proteins can be used to further improve the yields of recombinant proteins that are produced in the E. coli periplasm.

## MATERIALS AND METHODS

### Protein production.

E. coli Tuner(DE3) (Novagen) was used for the production of the scFv BL1 (23). Tuner(DE3) is a BL21(DE3)-derived strain that lacks the gene encoding β-galactosidase, which is the protein that is recognized by the scFv BL1. The gene encoding the scFv BL1 was fused at the 5′ end to the genetic information encoding either the DsbA signal peptide or the OmpA signal peptide. The genetic information encoding a His_6_ tag was fused to the 3′ end of the gene encoding the scFv BL1 to detect both the precursor and the processed form of the scFv BL1 by means of immunoblotting (13). DsbA-BL1 and OmpA-BL1 were produced from a pET28+-derived vector as described before (13). As a control, an “empty” vector was used as described before (13). Cells were grown aerobically at 30°C and 200 rpm in lysogeny broth (LB) medium (Difco) supplemented with 50 μg/ml kanamycin. Growth was monitored by measuring the optical density at 600 nm (OD_600_) with a UV-1601 spectrophotometer (Shimadzu). Protein production was induced for 4 h at an OD_600_ of ∼0.4 by adding 0.4 mM isopropyl-β-d-thiogalactopyranoside (IPTG) (final concentration).

### Subcellular fractionation.

Spheroplasts were generated and the periplasmic fraction isolated essentially as described before (14). In short, cells were incubated for 1 h in ice-cold osmotic shock buffer (75 optical density units [ODU]/ml in 20 mM Tris-HCl, pH 8.0, 2.5 mM EDTA, 30% [wt/vol] sucrose). Afterward, the cell suspension was diluted eight times using 20 mM Tris-HCl (pH 8.0) and incubated on ice for at least 30 min. Spheroplasts were pelleted at 10,000 × *g* for 10 min, and the supernatant representing the periplasm was concentrated using trichloroacetic acid (TCA) precipitation. All steps were carried out on ice or at 4°C. Protein concentrations were determined using the Pierce BCA protein assay kit (Thermo Fisher, USA).

### SDS-PAGE and immunoblotting.

Whole-cell lysates (0.1 OD_600_ units or 1 μg of protein) and periplasmic fractions (1 μg of protein) were analyzed by SDS-PAGE using standard polyacrylamide gels followed by immunoblotting as described before (17). The scFv BL1 was detected using a horseradish peroxidase (HRP)-conjugated α-His antibody (Thermo Fisher) recognizing its C-terminal His_6_ tag (13). The anti-SurA, -Skp, -OmpA, -SecY, -SecE, and -IbpB rabbit polyclonal antisera used are from our serum collection. The anti-DnaK mouse monoclonal antibody used is from Sigma, and the anti-σ^32^ monoclonal antibody is from NeoClone. The incubation with a primary antibody was, depending on its source, followed by incubation with either a secondary HRP-conjugated goat α-rabbit antibody (Bio-Rad) or a secondary HRP-conjugated goat α-mouse antibody (Bio-Rad). Proteins were visualized using the ECL system (GE Healthcare) according to the instructions of the manufacturer and a Fuji LAS-1000 charge-coupled-device (CCD) camera.

### BL1 activity assay.

The folding of the scFv BL1 was assessed by the recognition of its substrate, E. coli β-galactosidase, using a dot blot assay and whole-cell lysate as described previously (13). In short, different amounts of β-galactosidase were spotted onto a polyvinylidene difluoride (PVDF) membrane, and the membrane was subsequently incubated with cell lysate. To test if the correct disulfide bonds were formed in the scFv BL1, prior to the incubation with the immobilized β-galactosidase, cell lysates were reduced with 10% β-mercaptoethanol. Binding of the scFv BL1 was visualized using an HRP-conjugated α-His antibody (Thermo Fisher), the ECL system (GE Healthcare), and a Fuji LAS-1000 CCD camera.

### Mass spectrometry, protein identification, and quantitative proteomics.

E. coli cells were pelleted and subsequently lysed using a solubilization buffer containing 2% SDS, 50 mM NaCl, and 50 mM ammonium bicarbonate and probe sonication. Proteins were isolated using acetone precipitation and resuspended in 2% SDS. The protein concentrations were determined using the Pierce BCA protein assay kit (Thermo Fisher, USA). Ten micrograms of each sample was reduced, alkylated, and digested in solution according to Ytterberg et al. (24). After zip tipping (Merck Millipore Ltd., Ireland), 1 μg of each sample was separated using C_18_ reversed-phase (RP) columns coupled online to a liquid chromatography-tandem mass spectrometer (LC-MS/MS). The chromatographic separation was achieved using an acetonitrile (ACN)-water solvent system containing 0.2% formic acid. The gradient was set up as follows: 5 to 40% ACN for 89 min, 40 to 95% ACN for 5 min, and 95% ACN for 8 min, all at a flow rate of 300 nl/min. The samples were analyzed by linear trap quadropole (LTQ) Orbitrap Velos electron transfer dissociation (ETD) MS (Thermo Fisher Scientific, Germany). The spectra were acquired with a resolution of 60,000 in MS mode, and the top 5 precursors were selected for fragmentation using collision-induced dissociation (CID). Mass lists were extracted from the raw data using Raw2MGF v2.1.3 and combined into one file using Cluster MGF v2.1.1, programs part of the Quanti work flow (25). The data were searched against a concatenated version of the E. coli complete proteome database (UniProt accession number UP000000625), using the Mascot search engine v2.5.1 (Matrix Science Ltd., UK). The database included 12 non-E. coli proteins: the scFv BL1 without signal peptide, the scFv BL1 fused to the DsbA signal peptide, the scFv BL1 fused to the OmpA signal peptide, bovine serum albumin, bovine α-S1-casein, bovine β-lactoglobulin, the kanamycin resistance protein, T7 RNA polymerase, three keratins, and trypsin. In total, the database consisted of 8,628 sequences and 2,722,180 residues. The following parameters were used: tryptic digestion (maximum 2 miscleavages); carbamidomethylation (C) as fixed modification; deamidation (N/Q), oxidation (M), and pyroglutamate (Q) as variable modifications; 10 ppm as precursor tolerance; and 0.25 Da as fragment tolerance. The threshold for a 1% false-discovery rate (FDR) was calculated to a peptide score of 15.

The quantification was done using the Quanti work flow, which is a quantification software based on extracted ion chromatograms (25). In short, after searching the combined Mascot generic format (mgf) data against the E. coli complete proteome, the resulting data file and the raw files were uploaded into Quanti v2.5.4.3. The following parameters were used: score threshold, 15; mass tolerance, 10 ppm; minimum peptides/protein, 2; maximum allowed deviation in retention time, 5 min; rt order, 50; only “charge deconvolution” and “use best Mascot peptide.” The quantitative values were further processed by multiplying the values with the reference abundance and normalizing each sample to the median of the summed intensities for all of the samples. Pair comparisons between treatments were performed using the log_10_ transformed normalized protein intensities, and the log_2_ ratios were calculated from the mean of the two treatments compared, and Student's *t* test was used to estimate the *P* value for the comparison. To account for false positives during multiple testing, *q* values (i.e., FDR-adjusted *P* values) were also calculated (26).

## Supplementary Material

Supplemental file 1

Supplemental file 2
