# Supplementary material for: Posttranslational Targeting of a Recombinant Protein Promotes Its Efficient Secretion into the Escherichia coli Periplasm
Source: Appl Environ Microbiol. 2019 Jun 17;85(13):e00671-19. doi: 10.1128/AEM.00671-19 (PMC6581171; doi:10.1128/AEM.00671-19)
Supplement: Supplemental file 1 [file AEM.00671-19-s0001.pdf]

Supplementary Information

Post-translational targeting of a recombinant protein promotes its efficient secretion into the *E. coli* periplasm

A. Jimmy Ytterberg, Roman A. Zubarev,
Thomas Baumgarten

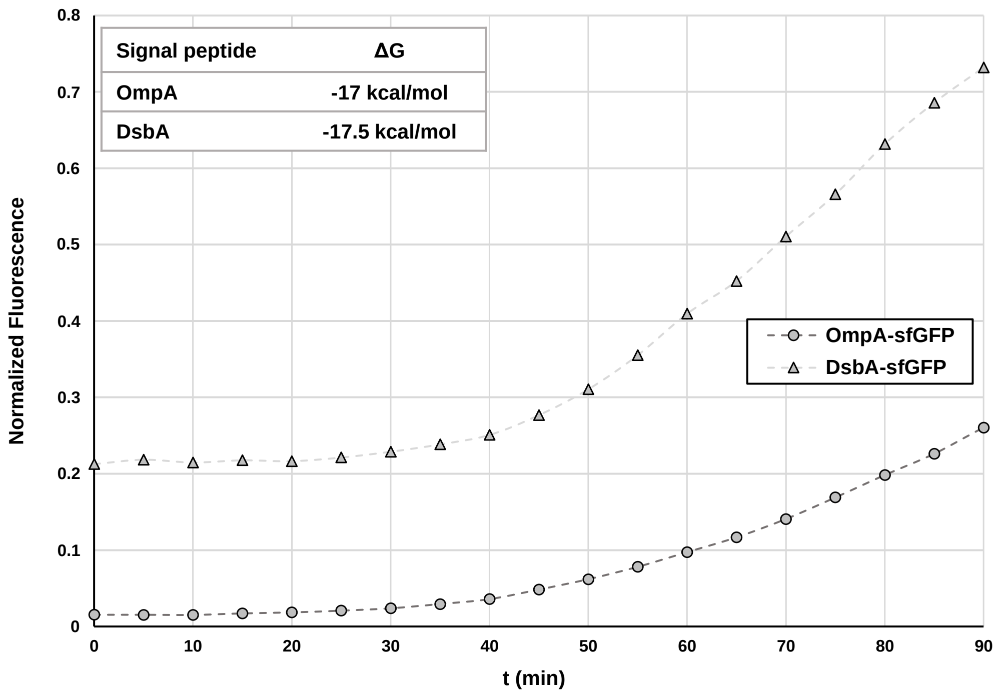

Figure S1. Impact of the signal peptide on protein production kinetics. The OmpA and the DsbA signal peptide was fused to sfGFP. Both fusion proteins were produced in *E. coli* BL21(DE3) and GFP fluorescence was monitored over time after protein production had been induced. The RNA folding predictor mFold was used to assess the potential structure formation around the translation initiation region starting from the ribosome binding site to the end of the respective signal peptide (insert).

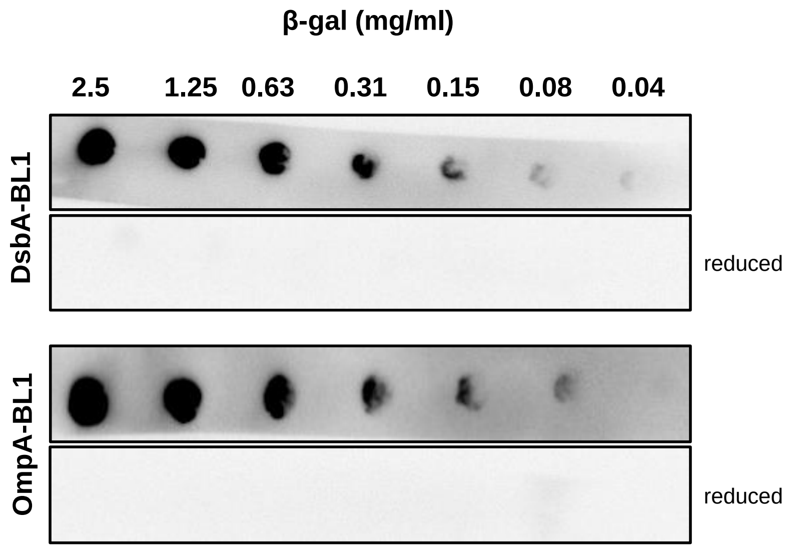

Figure S2. Producing the scFv BL1 in the periplasm. PVDF membranes containing decreasing amounts of β-galactosidase were incubated with whole cell lysates of cells producing either DsbA-BL1 or OmpA-BL1. Binding of the scFv BL1 present in whole cell lysates to β-galactosidase was detected using an α-His antibody recognizing the C-terminal His-tag of the scFv BL1. As a control, prior to the incubation with the PVDF membrane cell lysates were treated with β-mercaptoethanol, which breaks disulfide bonds present in the scFv BL1.

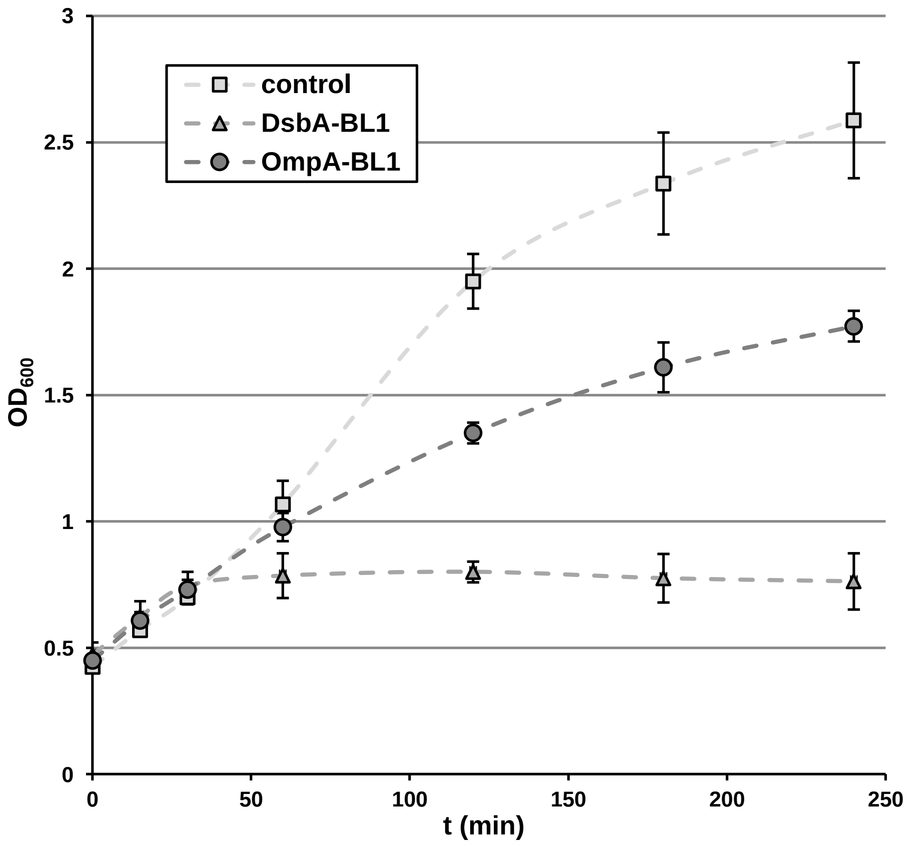

Figure S3. Impact of producing the secretory scFv BL1 on biomass formation. The production of either DsbA-BL1 or OmpA-BL1 was induced in *E. coli* Tuner(DE3) and biomass formation was monitored by measuring the OD_{600}.
